# Endothelium-Released Microvesicles Transport miR-126 That Induces Proangiogenic Reprogramming in Monocytes

**DOI:** 10.3389/fimmu.2022.836662

**Published:** 2022-02-18

**Authors:** Gemma Arderiu, Esther Peña, Anna Civit-Urgell, Lina Badimon

**Affiliations:** ^1^ Cardiovascular-Program, Institut de Recerca de l’Hospital de la Santa Creu i Sant Pau, IIB-Sant Pau Barcelona, Spain; ^2^ Ciber CV, Instituto Carlos III, Madrid, Spain

**Keywords:** monocytes, endothelial cell differentiation, microvesicles, miR-126, angiogenesis

## Abstract

We have recently shown that in ischemic tissue, the hypoxic endothelial cells (EC) release extracellular microvesicles (EMVs) that are rich in tissue factor (TF). These TF-EMVs induce monocyte (Mo) homing to the ischemic zone, their differentiation into EC-like cells, and the formation of new blood vessels increasing tissue perfusion. In addition to membrane proteins, EMVs contain noncoding RNAs that can modulate cellular signaling pathways in the recipient cells. Here, we have investigated whether miRNA contained into secreted EMVs may be transferred into Mo where they could modulate EC-like cell differentiation and angiogenic responses. Our results indicated that EMVs released from activated ECs contain high levels of miR-126 and that the levels are directly proportional to TF expression in EMVs. Interestingly, miR-126 is transferred to Mo when they are incubated with TF-EMVs. Increased levels of miR-126 in Mo do not promote EC-like cell differentiation but regulate angiogenesis by targeting several components of the VEGF pathway, as SPRED1 and PI3KR2. Our findings reveal that activated ECs secrete EMVs carrying miR-126, which can modulate Mo reprogramming of angiogenic genes.

## Introduction

During both homeostasis and inflammation, circulating monocytes (Mo), a subset of circulating white blood cells, leave the bloodstream, migrate into tissues, and differentiate into macrophage or dendritic cell populations (1). Recruitment of Mo is essential for the effective control and clearance of infection (2), but recruited Mo also contribute to the pathogenesis of inflammatory and degenerative diseases (3). In addition, it has been shown that Mo play an intricate role in angiogenesis (4). Mo can acquire endothelial features under proangiogenic stimulation. This is accompanied by a defined sequence of events involving the expression of endothelial differentiation markers, changes in cell morphology, and developing an endothelial cell-like (ECL) cell phenotype (5, 6).

The amount and the origin of microvesicles (MVs), as well as their contents, can vary during disease progression; it has been shown that after ischemia, endothelial cells (ECs) release MVs that promote postnatal neovascularization (7). Recent studies from our group have shown that tissue factor (TF) present in endothelial microvesicles (EMVs) induces Mo differentiation to ECs *in vitro* (8), and MVs rich in TF released by ECs after ischemic injury promote polarization of circulating blood Mo, recruitment into the damaged tissue, and differentiation into ECL cells promoting postischemic neovascularization and tissue repair (9).

MVs are important players in cell–cell communication, tissue homeostasis, cell differentiation, as well as organ development and remodeling (10–12). Their functions are not only due to the proteins present in their membranes but also to their content. Genetic material, mRNA and microRNA (miRNA), present in MVs and delivered to target cells has been recently recognized as a new mechanism of cell function regulation by intercellular communication (13, 14).

MiRNAs represent a class of small noncoding RNAs (about 20–25 nucleotides) that can bind to complementary target sites in mRNA molecules and repress translation or reduce mRNA stability (15). Many studies have revealed important roles of intracellular miRNAs in cellular differentiation (16, 17), and functional processes as angiogenesis (18–20), or inflammation (21, 22). MiR-126, the most abundant miRNA in ECs, has been reported to play a vital role in angiogenesis (23, 24), and it has been also found to inhibit vascular inflammation by targeting intercellular adhesion molecules that mediated leukocyte infiltration (25). Whether miR-126 can be shuttled *via* EMVs is not known.

In the current study, we have assessed whether EMVs can modulate Mo differentiation not only by protein transfer but also by sharing their miRNA content.

## Materials and Methods

### Cell Culture

Mouse RAW 264.7 monocyte cell line (RMC) (ATCC^®^ TIB-71™) and an immortalized human dermal microvascular endothelial cell line (HMEC-1) (26), a kind gift from the Centre of Disease Control (Atlanta, GA), were cultured in DMEM and MCDB-131 growth media, respectively, under standard cell culture conditions (37°C, 5% CO_2_). HMEC-1 were used throughout the investigation because in contrast to other cells (tumor cells), they do not either express TF or release EMVs spontaneously. Moreover, an immortalized HMEC-1 line retains the morphological, phenotypic, and functional characteristics of normal HMEC, and they can easily be genetically manipulated to overexpress or silence TF or miRNA.

### EMV Generation, Isolation, and Characterization

EMVs were generated from HMEC-1 as previously described in Arderiu el al (27). Briefly, 1 × 10^7^ cells were induced to migrate by injuring the cell monolayer with multiple scratches (more than 60% of wounded area) for 48 h to stimulate EMV release while ECs are migrating. After 48 h, the supernatant was collected and centrifuged at 1,500×*g* for 15 min to remove cell debris; afterward, it was centrifuged (20,000×*g* for 40 min) to pellet the EMVs. Pelleted EMVs were resuspended in serum-deprived sterile media.

In order to generate TF-upregulated EMVs (upTF-EMVs), TF-downregulated EMVs (siTF-EMVs), and empty vector EMVs (cnt-EMVs), HMEC-1 were transfected with their specific vector as described in Arderiu et al. (27).

The presence of phosphatidylserine on the surfaces of MVs allows the use of AnnexinV for the detection of MV by flow cytometry. After cellular activation, the process of MV formation is initiated, and intracellular calcium is increased which results in the rearrangement of phospholipid asymmetry with phosphatidylserine translocation. Isolated MVs were incubated with Annexin V CF-Blue (Immunostep, Salamanca, Spain) and an anti-TF antibody conjugated with Fluoroisothiocyanate (FITC) (Biomedica, Makati, Philippines). Samples were diluted with Annexin V Binding Buffer (BD Biosciences, Franklin Lakes, NJ, USA) before being immediately analyzed on a FACSCantoII flow cytometer (BD Biosciences). CD63 (CD63-APC Vio770) and CD47 (CD47-PE) were also used to identify MVs. TF-bearing MPs were identified and quantified based on their FSC/SSC characteristics according to their size, binding to Annexin V, and reactivity to anti-TF antibody. MV gate limits were established into the following two criteria: (1) calibration using a Flow-Check Size Range Calibration Kit (Polysciences) and (2) using platelet-derived microparticles as positive control. The lower detection limit was placed as a threshold above the electronic noise of our flow cytometer. Data were analyzed with FACSDiva software (BD). The concentration (number of Annexin V and TF-positive MVs per microliter of supernatants) was determined according to Nieuwland’s procedure (28), based on sample’s volume, flow cytometer’s flow rate, and the number of fluorescence-positive events. Background cell autofluorescence was assessed by omission of antibody.

### EMVs Quantification

Microvesicle concentration was determined as described in the study by Arderiu et al. (27). Briefly, microvesicles were quantified using the Zymuphen microparticle determination kit (Hyphen BioMed, Neuville-sur-Oise, France). Experiments were performed with 100,000 MVs/ml because we calculated that it corresponds to a TF concentration of 100 pM in upTF-EMVs. TF concentration is in the range of that found in the plasma of patients with cardiovascular disease (50 to 200 pM) (29).

### Monocyte-EMV Interaction

RMCs were labeled with living cell fluorescent membrane dye PKH26 Red Fluorescent linker Kit (Sigma, St. Louis, MO, USA) and treated with EMVs labeled with living cell fluorescent membrane dye PKH17 Green Fluorescent linker Kit (Sigma) (at a ratio of 1:5). Interaction was monitored by time-lapse video microscopy at 15-min intervals. Cells were viewed using a PL APO ×20/0.7 Multi-immersion CS. Images were acquired, digitalized, and processed with Leica Software TCS-AOBS. Leica Software was used to perform and analyze the cytofluorograms.

### Monocyte Differentiation Into Endothelial Cell-Like Cells

RMCs were seeded on plates covered with 10 µg/ml of fibronectin and cultured with DMEM or with endothelial media supplemented with cnt-EMVs, with upTF-EMVs, or with siTF-EMVs. Cells were resupplied with fresh EMV-containing medium every 2–3 days and cultured for a total of 8–10 days. At this time, vascular endothelial cell markers (VECMs) were analyzed by flow cytometry.

### Raw Transfection With miRNA Mimics

RMCs were transfected with 100 nM of mirVarna hsa-miR-126-3p mimic or 100 nM mirVana-negative control (AMBION by Life Technologies Corporation, Carlsbad, CA, USA). Cells were transfected using Lipofectamine RNAiMAX Reagent (Life Technologies Corporation) according to the manufacturer’s protocol.

### Real-Time Quantitative Reverse-Transcriptase Polymerase Chain Reaction

For miRNA expression, total RNA extraction was performed with the mirVana™ miRNA Isolation Kit (Life Technologies Corporation) according to the manufacturer’s instructions. RNA Spike-In Kit (Exiqon, Vedbæk, Denmark) was used to determine the quality of the sample. cDNA was synthesized by TaqMan™ Advanced miRNA cDNA Synthesis Kit (Life Technologies). miRNA levels were quantified by real-time PCR using hsa-miR-126-3p and internal normalized control hsa-miR-186-5p (Applied Biosystems).

For gene expression, RNA from cell lysates was extracted by RNeasy isolation kit (Qiagen, Hilden, Germany) and reverse transcribed. mRNA levels were analyzed by real-time PCR. Assays were used for CDH5 (Ve Cadherin) (00486938_m1); vWF (01182962_m1); NOS3 (eNOS) (00435217_m1); F3 (TF) (00438853_m1); SPRED1 (Mm00486938_m1); PI3KR2 (Mm00435694_m1); VCAM-1 (Mm01320970_m1); IL-12p40 (Il2b) (Mm99999067_m1); IL-23p19 (Il23a) (Mm00518984_m1); IL-beta1 (Il1b) (Mm00434228_m1); and TNFalpha (tnf) (Mm00443258_m1) (Life Technologies). 18S was used as endogenous control.

### Immunoblot Analysis

A total of 10% SDS-PAGE gel was run and transferred to nitrocellulose membranes. After blocking for nonspecific binding, Western blots were probed with a monoclonal anti-TF antibody (American Diagnostica, Stamford, CT, USA), rabbit monoclonal anti-phospho ERK 1/2 (p44/42 Thr202/Tyr204) (D13.144E), monoclonal anti-ERK 1/2 (137F5), rabbit monoclonal anti-phospho AKT (ser473), and rabbit monoclonal anti-AKT1 (C73H10) (Cell Signaling, Danvers, MA, USA); rabbit polyclonal anti-SPRED1 (Novus Biologicals, St. Louis, MO, USA); and rabbit polyclonal anti-PI3K p85 beta (PI3KR2) followed by anti-mouse-HRP-conjugated secondary antibody at 1:2,000. Excess of antibody was removed by extensive washing and blots were developed by ECL system (Amersham Biosciences, Amersham, UK). The membranes were then stripped and treated with polyclonal anti-β-actin antibody (1:1,000) (Abcam Inc., Cambridge, MA, USA), followed by donkey antirabbit-HRP at 1:5,000 and detected by ECL system. Band densities were determined with the ChemiDoc™ XRS system (Bio-Rad, Hercules, CA, USA) in chemiluminescence detection modus and Quantity-One software (Bio-Rad).

### Flow Cytometry Analysis

Mo-differentiated ECL cells were removed from plates with PBS/EDTA 0.53 mM. Levels of VE-Cadherin, vWF, and e-NOS were analyzed by FACS. Briefly, cells were stained with anti-VE-Cadherin Alexa Fluor 488 (eBioscience, San Diego, CA, USA) and anti-von Willebrand Factor (Abcam) with Alexa Fluor 488 donkey anti-sheep IgG as a secondary antibody. To analyze eNOS expression, cell suspension were permeabilized with INTRACELL (Immunostep) and incubated with anti-eNOS-PE antibody (BD Phosflow). Samples incubated with the same final concentration of isotype-matched antibody were used as a negative control.

### Statistical Analysis

Results were expressed as mean ± standard error of the mean (SEM), and the number of experiments is indicated in the figure legends. Values were tested for statistical differences by the parametric analysis of variance (ANOVA) or Student’s *t*-test for independent measures and the Tukey’s HSD *post-hoc* test when data had a normal distribution and the nonparametric analysis of variance test Mann–Whitney *U* or Kruskal–Wallis *H* for independent measures when data did not have a normal distribution. The statistical software package IBM^®^SPSS Statistics 22 (SPSS Inc., Chicago, IL, USA) was used for statistical analyses. Differences were considered statistically significant when *p* < 0.05.

## Results

### EMVs Transfer miRNAs to Recipient Monocytic Cells

**Figure 1:**
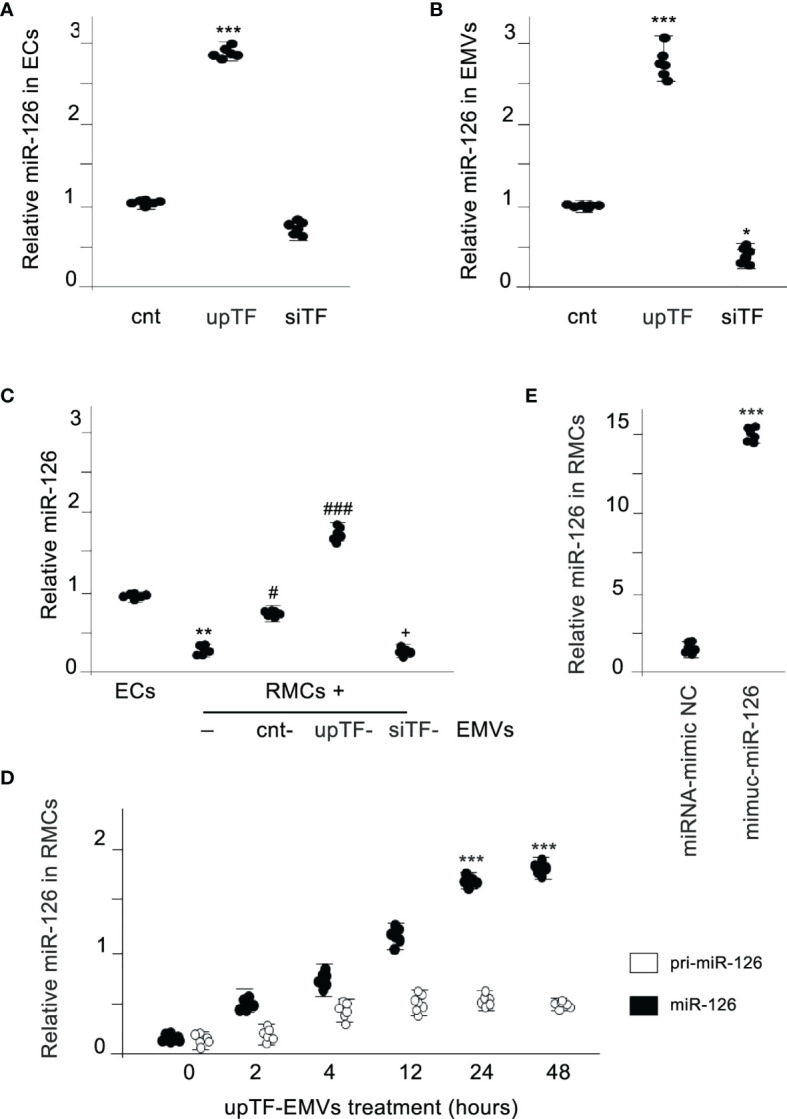
MiR-126 is transferred from EMVs to monocytic cells. **(A)** Expression of TF in ECs results in a dependent expression of miR-126 in ECs. MiR-126 expression levels were analyzed in ECs transfected with control vector (cnt), TF overexpression vector (upTF), or silencing TF (siTF) by qRT-PCR. Values are expressed as mean ± SEM. Statistical analysis was performed by ANOVA followed by Tukey’s *post-hoc* test. ^***^ *p* < 0.001 vs. cnt (*N* = 6). **(B)** qRT-PCR was used to assess the expression of miR-126 in EMVs released from activated control ECs, TF overexpressing ECs (upTF), or TF-silenced ECs (siTF). Values are expressed as mean ± SEM. Statistical analysis was performed by ANOVA followed by Tukey’s *post-hoc* test. ^*^ *p* < 0.05 and ^***^ *p* < 0.001 vs. cnt (*N* = 6). **(C)** MiR-126 expression analyzed by qRT-PCR in endothelial cells (EC) and RMCs treated for 24 h without or with EMVs released from control ECs, TF overexpressing ECs (upTF) or TF silenced ECs (siTF). Values are expressed as mean ± SEM. Statistical analysis was performed by ANOVA followed by Tukey’s *post-hoc* test. ^**^ *p* < 0.01 vs. ECs, ^#^ *p* < 0.05, and ^###^ *p* < 0.001 vs. RMCs without EMVs and ^+^ *p* < 0.05 vs. RMCs treated with cnt-EMVs (*N* = 6). **(D)** Kinetics of mature miR-126 and pri-miR-126 expression after treatment of RMCs with upTF-EMVs. Statistical analysis was performed by ANOVA followed by Tukey’s *post-hoc* test. ^***^ *p* < 0.001 vs. time 0. (*N* = 6). **(E)** Expression of miR-126 in RMCs treated with EMVs released from ECs transfected with miRNA-mimic-negative control (NC) or mimic-miR-126. Values are expressed as mean ± SEM. Statistical analysis was performed by ANOVA followed by Tukey’s *post-hoc* test. ^***^ *p* < 0.001 vs. cnt (*N* = 6).

We analyzed whether miR-126-3p was present in released EMVs and whether there was a relation with the TF content in the EMVs. Comparison of microvascular ECs^empty vector^ (ECs), microvascular ECs^TF-upregulated^ (ECs^upTF^), or microvascular ECs^TF-downregulated^ (ECs^siTF^) and the released EMVs (**Supplementary Figures S1A, B** showing TF expression) revealed that expression of miR-126-3p was directly associated to TF expression. Large amounts of miR-126-3p were detected in cells with high TF expression, and on the contrary, a negligible amount of miR-126-3p was detected in TF-silenced cells (**Figure 1A**). Similar results were obtained when miR-126-3p was analyzed in the released EMVs (**Figure 1B**). miR-126-3p is abbreviated as miR-126 in the subsequent experiments.

We analyzed afterwards whether miR-126 could be transferred *via* released EMVs to monocytic cells. **Figure 1C** shows that RAW monocytic cells (RMCs) did not express miR-126 compared with ECs; however, when RMCs were treated with EMVs, miR-126 increased significantly in these RMCs. Importantly, miR-126 levels rapidly increased, but the primary miR-126 transcript was not significantly induced, indicating a transfer from EMVs but not transcriptional induction (**Figure 1D**). In addition, transfection of miR-126 mimics into ECs (**Supplementary Figure S1C**) resulted in an increase in miR-126 in EMVs and in the treated RMCs (**Figure 1E**).

Taken together, these results provide evidence that in cells, miR-126 expression is proportional to TF expression and TF determines the level of miR-126 released in EMVs and, that miR-126 is directly transferred from EMVs to monocytic cells.

### MiR-126 Does Not Induce Vascular Endothelial Cell Markers in Monocytes

**Figure 2:**
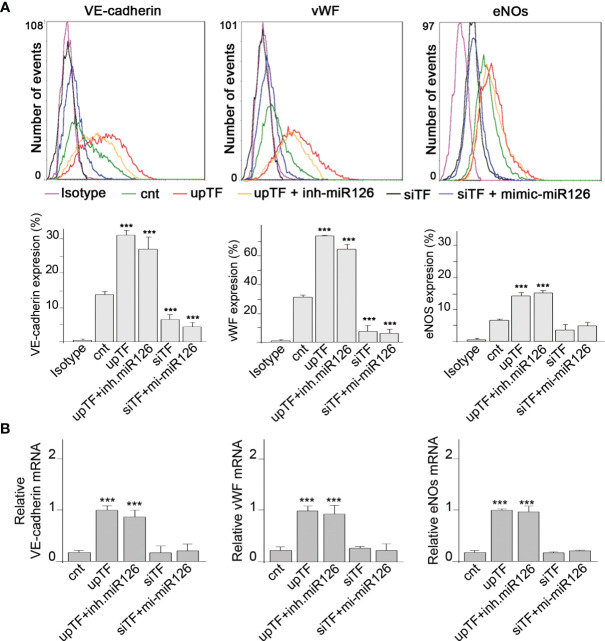
MiR-126 in EMVs does not induce vascular endothelial cell markers in RMCs. **(A)** Expression protein of VE cadherin, von Willebrand factor (VWF), and eNOS was analyzed by fluorescence-activated cell sorting (FACS) analysis in RMCs after 8 days of treatment with EMVs obtained from ECs transfected with control (empty vector) (cnt), TF overexpression vector with or without inhibitor miR-126, or TF silencing with or without mimic-miR-126. Unspecific IgG antibody was used as a control. Histograms show analysis from 6 experiments. Values are expressed as mean ± SEM. Statistical analysis was performed by ANOVA followed by Tukey’s *post-hoc* test. ^***^ *p* < 0.001 vs. RMCs treated with cnt-EMVs. **(B)** mRNA expression of VE-cadherin, von Willebrand factor (VWF), and eNOS was analyzed by qRT-PCR in RMCs after 8 days of treatment with EMVs obtained from ECs transfected with control (cnt), TF overexpression vector with or without inhibitor miR-126, or TF silencing with or without mimic-miR-126. Values are expressed as mean ± SEM. Statistical analysis was performed by ANOVA followed by Tukey’s *post-hoc* test. ^***^ *p* < 0.001 vs. RMCs treated with cnt-EMVs.

**Figure 3:**
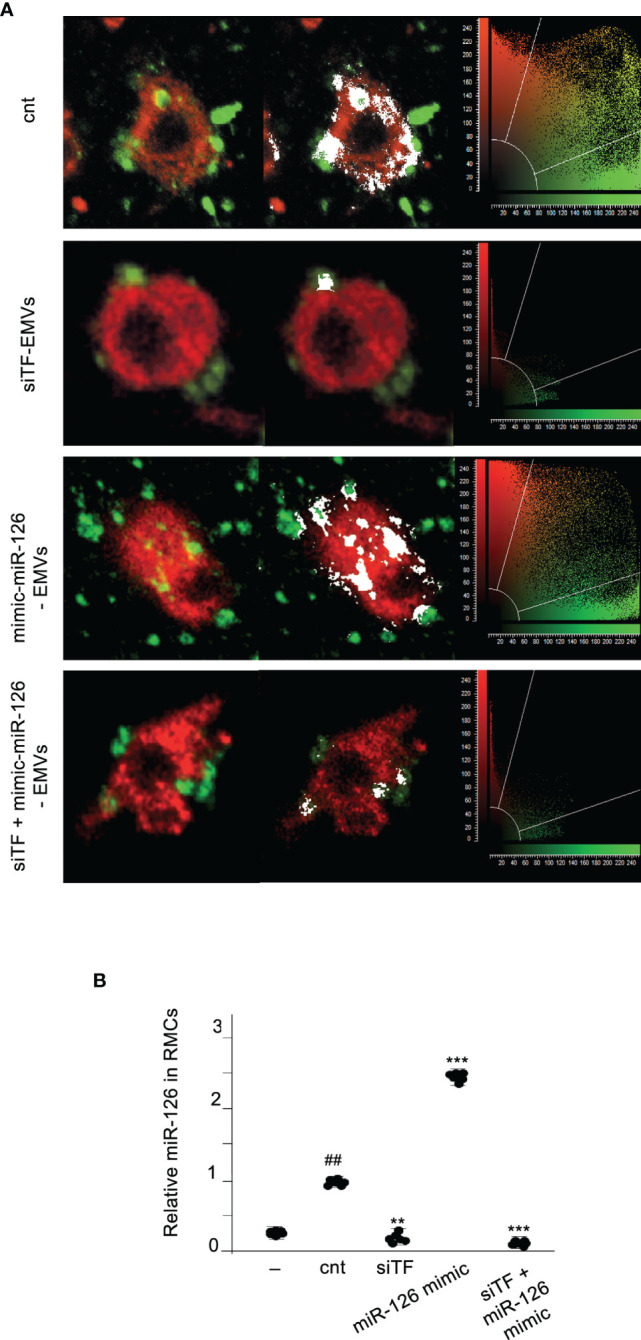
TF is needed for transferring MiR-126 from EMVs to monocytic cells. **(A)** Immunofluorescence staining of 24-h cultures of RMCs (stained with PKH67 Green Fluorescence Cell Linker) and EMVs (stained with PKH67 Red Fluorescence Cell Linker) isolated from ECs transfected with empty vector (cnt), TF silenced (siTF), mimic miR-126 (miR-126 mimic), and TF silenced and mimic miR-126 (siTF + miR-126 mimic). Colocalization is shown in white. Cytofluorograms are shown together with confocal images. **(B)** qRT-PCR was used to assess the expression of miR-126 in RMCs cultured for 24 h without (−) or with EMVs released from activated control ECs, TF-overexpressing ECs (upTF), or TF-silenced ECs (siTF). Values are expressed as mean ± SEM. Statistical analysis was performed by ANOVA followed by Tukey’s *post-hoc* test. ^##^ *p* < 0.01 vs. RMCs without EMVs (−), ^**^ *p* < 0.01 and ^***^ *p* < 0.001 vs. RMCs with EMVs from ECs transfected with empty vector (cnt) (*N* = 6).

We had demonstrated that TF present in EMVs induces monocyte differentiation into ECL cells (9). Here, we analyzed whether miR-126 contained into TF-rich EMVs contributed to monocyte differentiation into ECL cells. TF and miR-126 expression were regulated in ECs using mimics and inhibitors, and their released EMVs (**Supplementary Figures S2**) were used to treat RMCs during 8 days. Vascular endothelial cell markers (VECMs) were analyzed by flow cytometry and RT-PCR. **Figure 2** shows that VE-cadherin, vWF, and eNOs (VECMs) were increased in RMCs inducing monocyte differentiation into ECL cells only when TF was present in EMVs. Inhibition of miR-126 did not modify VECM expression. However, VECM expression was decreased when TF was silenced and was not rescued with mimic-miR-126. In previous studies, we had demonstrated that interaction of EMVs and RMCs needs the presence of TF in EMVs and β1-integrin on RMCs (8); in addition, TF promotes transcriptional regulation and intracellular signaling that mediates Mo to ECL cell differentiation (9). **Figure 3A** shows that when TF was silenced in ECs and consequently EMVs did not present TF in their surfaces, EMV were not able to interact with RMCs and mimic-miR-126 was not transferred into RMCs (**Figure 3B**).

**Figure 4:**
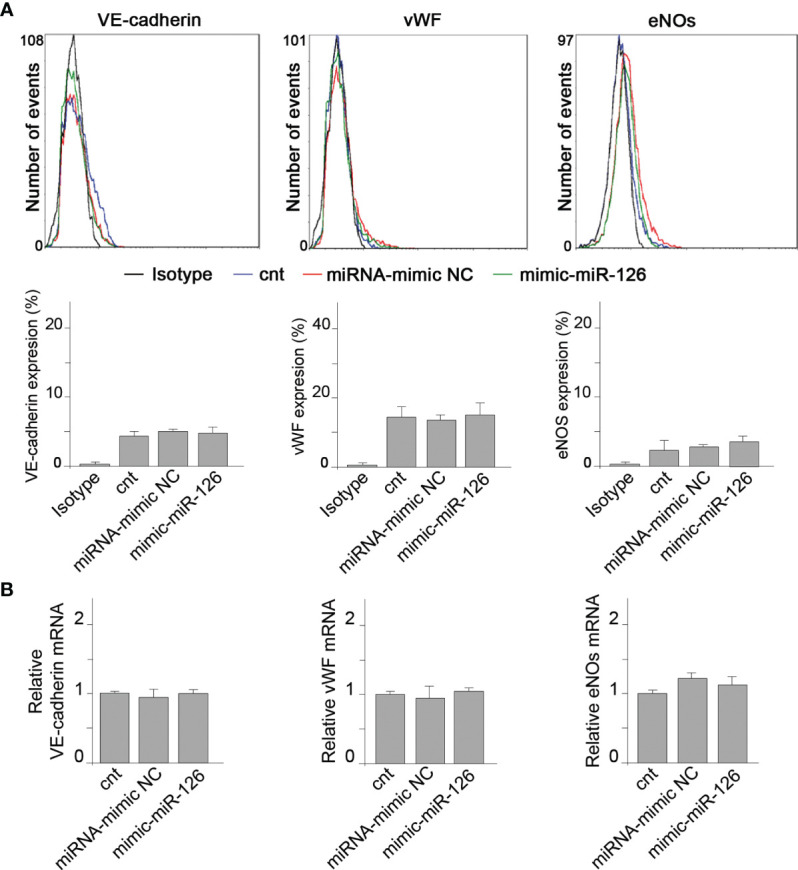
MiR-126 in RMCs does not induce vascular endothelial cell marker expression. **(A)** Expression of VE-cadherin, von Willebrand factor (VWF), and eNOS was analyzed by fluorescence-activated cell sorting (FACS) analysis in RMCs not transfected (cnt) or transfected with miRNA mimic-negative control (NC) or mimic-miR-126. Unspecific IgG antibody was used as a control. Histograms show analysis from 6 experiments. Values are expressed as mean ± SEM. Statistical analysis was performed by ANOVA followed by Tukey’s *post-hoc* test. **(B)** mRNA expression of VE-cadherin, von Willebrand factor (VWF), and eNOS was analyzed by qRT-PCR in RMCs not transfected (cnt) or transfected with miRNA mimic-negative control (NC) or mimic-miR-126. Values are expressed as mean ± SEM. Statistical analysis was performed by ANOVA followed by Tukey’s *post-hoc* test (*N* = 6).

We further analyzed the direct effect of miR-126 in RMCs. RMCs were transfected with mimic-miR-126 (**Supplementary Figures S3**) and VECMs were analyzed by flow cytometry and quantitative reverse-transcriptase polymerase chain reaction (qRT-PCR). **Figure 4** shows that overexpression of miR-126 in RMCs did not induce differentiation into ECL cells because there was no induction of VECM expression.

These results indicate that miR-126 present in EVMs is not a direct regulator of VECM expression in monocytic cells.

### MiR-126 Regulates Downstream Angiogenic Target Genes

**Figure 5:**
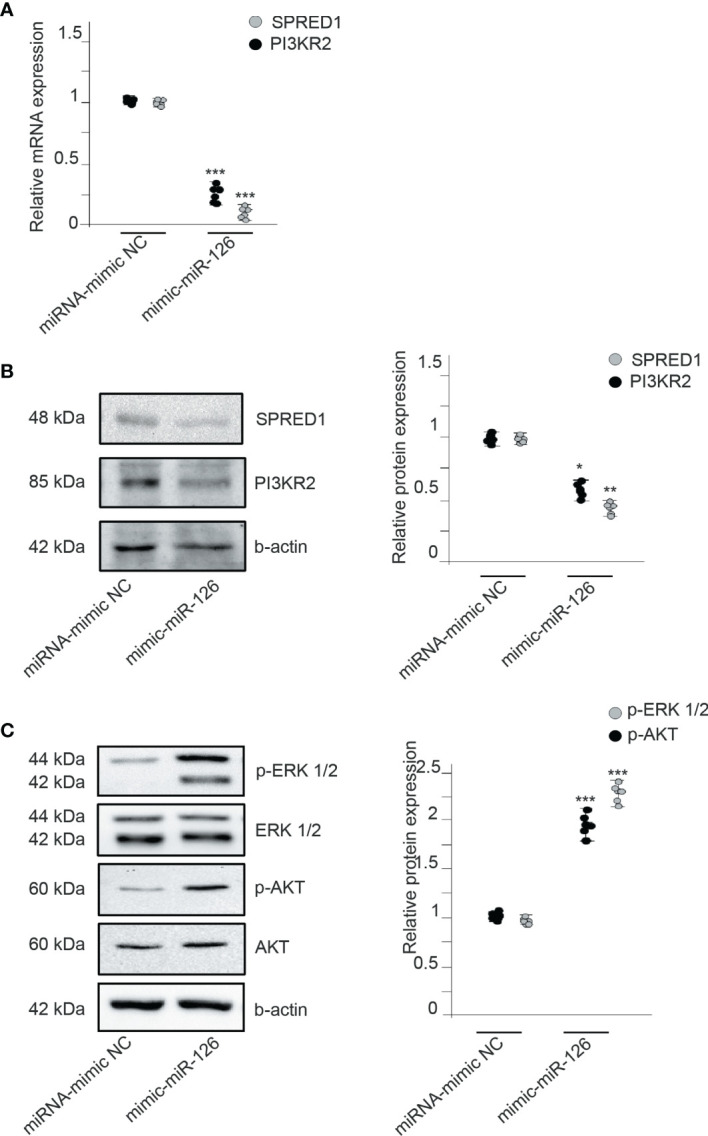
MiR-126 regulates VEGF signaling by repressing SPRED1 and PI3KR2. **(A)** Relative expression of SPRED1 and PI3KR2 mRNA levels in RMCs transfected with miRNA-mimic NC or mimic-miR-126. Values are expressed as mean ± SEM. Statistical analysis was performed by ANOVA followed by Tukey’s *post-hoc* test. ^***^ *p* < 0.001 vs. miRNA-mimic NC (*N* = 6). **(B)** SPRED1 and PI3KR2 protein levels in RMCs transfected with miRNA-mimic NC or mimic-miR-126. Blots are representatives from 6 independent experiments. Values of relative protein levels normalized to β-actin are expressed as mean ± SEM. Statistical analysis was performed by ANOVA followed by Tukey’s *post-hoc* test. ^*^ *p* < 0.05 and ^**^ *p* < 0.01 vs. respective miRNA-mimic NC. **(C)** Western blot analysis of phospho-ERK 1/2, ERK 1/2, phospho-AKT Ser473, and AKT protein in RMCs transfected with miRNA-mimic NC or mimic-miR-126. To test for equal loading, Western blots were reprobed by β-actin (*N* = 6). Quantitative analysis of phospho-ERK 1/2 relative to ERK 1/2 and phospho-AKT Ser473 relative to AKT in each condition. Values are expressed as mean ± SEM. Statistical analysis was performed by ANOVA followed by Tukey’s *post-hoc* test. ^***^ *p* < 0.001 vs. respective miRNA-mimic NC (*N* = 6).

We searched by data mining for potential direct gene targets of miR-126. Sprouty-related EVH1 domain containing 1 (SPRED1), phosphoinositide 3-kinase regulatory subunit p85 beta (PIK3R2), and vascular cell adhesion molecule 1 (VCAM1) are three validated target genes of miR-126 (25). Previous studies found that miR-126 promotes angiogenesis by repressing SPRED1 and PIK3R2, and inhibits vascular inflammation by repressing VCAM1 expression posttranscriptionally. RMCs transfected with miRNA-mimic NC or miR-126 mimic showed an inversely regulated expression of mRNA levels of SPRED1 and PIK3R2 (**Figure 5A**). Protein expression was also assessed by obtaining similar results as mRNA expression; increased levels of miR-126 decreased levels of SPRED1 and PIK3R2 proteins (**Figure 5B**).

We also analyzed the underlying mechanisms of miR-126 in angiogenesis; we studied the phosphorylation of ERK1/2 and AKT1, related to SPRED1 and PIK3R2, respectively. miR-126 overexpression significantly induced the expression levels of p-AKT and p-ERK compared with the control RMCs (**Figure 5C**). To discriminate the involvement of miR-126 and not of TF, RMCs were treated with upTF-EMVs obtained from miR-126-inhibited ECs. Results showed that in RMCs treated with upTF and inhibitor miR-126, EMVs did not show changes in SPRED1 and PIK3R2 (**Supplementary Figure S4**).

Therefore, miR-126 transferred to RMCs negatively represses the regulators of VEGF pathways SPRED1 and PIK3R2.

### MiR-126 Can Suppress Proinflammatory Monocytic Cell Activation

**Figure 6:**
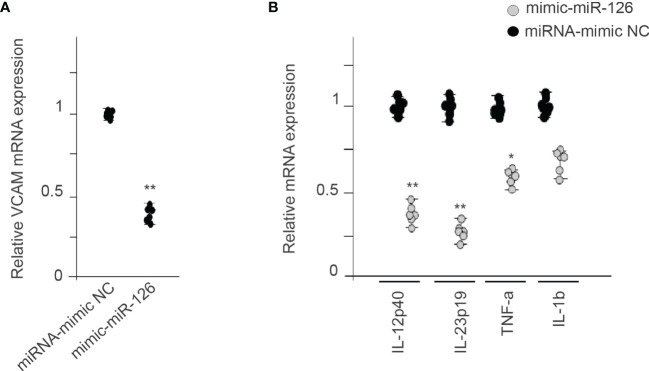
MiR-126 in RMCs reduces proinflammatory genes. **(A)** Relative expression of VCAM1 mRNA levels in RMCs transfected with miRNA-mimic NC or mimic-miR-126. Values are expressed as mean ± SEM. Statistical analysis was performed by ANOVA followed by Tukey’s *post-hoc* test. ^**^ *p* < 0.01 vs. miRNA-mimic NC (*N* = 6). **(B)** qRT-PCR analysis of proinflammatory genes in RMCs transfected with miRNA-mimic NC or mimic-miR-126. Values are expressed as mean ± SEM. Statistical analysis was performed by ANOVA followed by Tukey’s *post-hoc* test. ^*^ *p* < 0.05 and ^**^ *p* < 0.01 vs. miRNA-mimic NC (*N* = 6).

MiR-126 also inhibits vascular inflammation by suppressing VCAM1 expression posttranscriptionally (30). **Figure 6A** shows that expression of miR-126 in RMCs decreases VCAM1 mRNA levels compared with the miRNA-mimic NC group. We also analyzed different proinflammatory genes, showing that miR-126 expression in RMCs decrease IL-12p40, IL-23p19, IL-β1, and TNF-α (**Figure 6B**).

## Discussion

Monocytes, important members of the innate immunity system, play an intricate role in angiogenesis. TF released within EMVs secreted by ischemic tissue induces Mo transformation and differentiation into ECL cells to promote tissue repair. Here, we have further investigated the mechanisms of EMV-induced Mo differentiation into ECL cells. We show that miR-126 contained in TF-rich EMVs may be transferred into Mo and increases their angiogenic properties. Upregulation of miR-126 in Mo induces downregulation of mRNA and protein levels of three of its target genes, SPRED1, PI3KR2, and VCAM1, increasing angiogenic properties and attenuating inflammation.

Mo are continually exposed to MVs in the circulation, but little is known of the functional consequences of MV uptake. In physiologic conditions, quiescent endothelium secretes few EMVs; however, in chronic inflammatory states, high levels of EMVs are released and are preferentially taken up by monocytes compared with neutrophils and lymphocytes, leading to monocyte activation (31). Endothelial dysfunction and activation, common in cardiovascular desease, likely contributes to alterations in the levels and types of EMVs released in the circulation, ultimately influencing their composition, function, and effects (32). EMVs relesed by active ECs contribute to the polarization of circulating Mo and recruitment into ischemic tissue after femoral artery ligation. We have described that these effects are induced by TF present in the EMV surface (9).

Here, our findings provide a new functional role of the cross-talk between Mo-ECs through EMVs, orchestrating vascular inflammation and angiogenesis in ischemic diseases. Increasing evidence suggests that EMV effects do not only depend on the proteins expressed on their surface but also on the EMV-miR content (33). In this way, it has been described that miR-126-3p, which is among the most abundant miRNAs in ECs, is present in EMVs and play multiple roles in cell-to-cell communication (34). Our results show that miR-126 is found in the EMVs released by activated ECs, and the miR-126 content in those EMVs is directly proportional to the levels of TF. MiR-126 is easily transferred to Mo.

miR-126 is a miRNA highly enriched in ECs (30). It is located in intron 7 of the EGF-like domain 7 (EGFl7) gene and is regulated by the transcription factors Ets1 and Ets2 (35). Ets1 is expressed in Mo after treatment with TF-rich EMVs; however, we found here increased levels of miR-126 but not pri-miR-126. It is possible that these results are due to the short study time, 24 h after treatment.

MiR-126 appears to play a critical role in angiogenesis and inflammatory responses. Mice treated with an antagomir-126 exhibit a markedly reduced angiogenic response after hind limb ischemia (36). MiR-126 promotes stabilization and maturation of growing blood vessels by regulating angiopoietin-1 signaling and by repressing the p21-activated kinase 1 gene (37, 38). MiR-126 also enhances angiogenesis repressing negative regulators of the VEGF pathway; it promotes blood vessel formation by repressing the expression of SPRED1 and PI3KR2 (23, 24). Here, we show that the proangiogenic actions of miR-126 have been transferred by EMVs to recipient Mo, where miR-126 keeps repressing SPRED1 and PI3KR2 expression.

SPRED1 is the only member of the SPRED family that contains a predicted target sequence for miR-126. SPRED1 regulates ERK activation and blocks cell proliferation and migration in response to growth factor signaling. SPRED inhibited the activation of MAP kinase by suppressing phosphorylation and activation of Raf-Ras (39). PI3KR2 is also a target of miR-126 (40). In vascular endothelial cells, miR-126 could negatively regulate the PI3K/Akt signaling pathway by targeting PIK3R2 (23). PI3KR2 negatively regulated the activity of PI3 kinase, a kinase important in the AKT pathway, which is related to an antiapoptotic effect (41).

Here, we show that EMV interaction with Mo induced angiogenesis by: (1) differentiation of Mo into ECL cells through the expression of VECMs, an activity TF dependent and (2) activation of signaling pathways directly related to the angiogenic process by repressing SPRED1 and PI3KR2 expression, which negatively regulate MAK kinase and AKT signaling, respectively.

MiR-126 not only plays a role in angiogenesis. It also has anti-inflammatory activity by regulation of VCAM1. Suppressing VCAM1 expression by miR-126, decreases leukocyte interaction with endothelial cells and attenuates vascular inflammation after injury (30). MiR-126 decreases VCAM1 in Mo and other proinflammatory genes. MiR-126 functions as a regulator of Mo-mediated inflammatory responses in primary monocytes from chronic HIV-1 patients by targeting CYLD (42). Overexpression of MiR-126-3p in rat chondrocyte promotes migration and proliferation and suppresses apoptosis and IL-1β, IL-6, and TNF-α expression (43). Here, we show that EMV transfer of miR-126 into Mo downregulates inflammatory genes as well.

As a limitation to our study, we should mention the use of a mouse monocytic cell line to describe the mechanisms. Although our study is based on well-established and characterized cell lines, our results may need confirmation in human cell lines to be clinically relevant. Interestingly, previous reports have shown that results obtained in RAW 264.7 cells are reproducible in human cell lines, such as THP-1 (44, 45). Further studies in 3-D basement membrane assays and *in vivo* are needed to demonstrate the transdifferentiation of monocytes into functional ECs able to form tube-like structures. Finally, the direct relationship of miR-126 expression and TF expression in ECs, although very interesting, falls out of the scope on this manuscript, but the mechanism will be addressed in further studies.

Previously, we had demonstrated that ECs in ischemic tissue release TF-rich EMVs, and these EMVs interact with Mo through TF-β1 integrin-promoting ETS1 transcription factor activation and expression of VECMs leading to differentiation of Mo into ECL cells (8, 9). Here, we further demonstrate that TF-rich EMVs also transfer miR-126 contained into the EMVs and improve Mo-angiogenic properties through transcriptional regulation. Taken together, our studies show a novel crosstalk between ECs and Mo that is mediated *via* secreted EMVs that contain proangiogenic miRNAs.

## Data Availability Statement

The original contributions presented in the study are included in the article/**Supplementary Material**. Further inquiries can be directed to the corresponding author.

## Author Contributions

GA and LB conceived and designed the study. GA, EP, and AC-U performed experimental work and analyzed the data. GA, EP, and AC-U wrote the original draft. LB revised the manuscript. Founding acquisition was performed by GA and LB. All authors listed have made a substantial, direct, and intellectual contribution to the work and approved it for publication.

## Funding

This work was supported by the Spanish Cardiovascular Network of Cell Therapy (RedTerCel RD16/0011/018 to LB), Ciber CV (CB16/11/00411 to LB), and PI20/01517 to GA from the Instituto Salud Carlos III. PID2019-107160RB-I00 to LB from Plan Nacional de Salud (SAF2016-76819-R to LB) from the Spanish Ministry of Science and Innovation. All grants were cofinanced by the European Union Funds, Fondo Europeo de Desarrollo Regional (FEDER) “Una manera de hacer Europa”.

## Conflict of Interest

The authors declare that the research was conducted in the absence of any commercial or financial relationships that could be construed as a potential conflict of interest.

## Publisher’s Note

All claims expressed in this article are solely those of the authors and do not necessarily represent those of their affiliated organizations, or those of the publisher, the editors and the reviewers. Any product that may be evaluated in this article, or claim that may be made by its manufacturer, is not guaranteed or endorsed by the publisher.

## Abbreviations

Cnt-ECs, endothelial cells transfected with empty vector; cnt-EMVs, endothelial microvesicles released by endothelial cells transfected with empty vector (control of transfection); ECs, endothelial cells; ECL, endothelial cell-like; EMVs, endothelial microvesicles; mECs, microvascular endothelial cells; miR, microRNA; Mo, monocytes; MVs, microvesicles; RMCs, raw monocytic cells; siTF-ECs, endothelial cells with silenced tissue factor (siRNA); siTF-EMVs, endothelial microvesicles released by endothelial cells with silenced tissue factor (siRNA); TF, tissue factor; TF^+^EMVs, tissue factor-rich endothelial microvesicles; TF^-^EMVs, tissue factor-poor endothelial microvesicles; upTF-ECs, endothelial cells overexpressing tissue factor; upTF-EMVs, endothelial microvesicles released by endothelial cells overexpressing tissue factor; VECMs, vascular endothelial cell markers.
