# Supplementary material for: Endothelium-Released Microvesicles Transport miR-126 That Induces Proangiogenic Reprogramming in Monocytes
Source: Front Immunol. 2022 Feb 18;13:836662. doi: 10.3389/fimmu.2022.836662 (PMC8894588; doi:10.3389/fimmu.2022.836662)
Supplement: Supplementary file 1 [file DataSheet_1.pdf]

Supplemental material

Endothelium released microvesicles transport miR-126 that induces pro-angiogenic reprogramming in monocytes

Gemma Arderiu, Esther Peña, Anna Civit-Urgell and Lina Badimon

Supplementary Figure 1

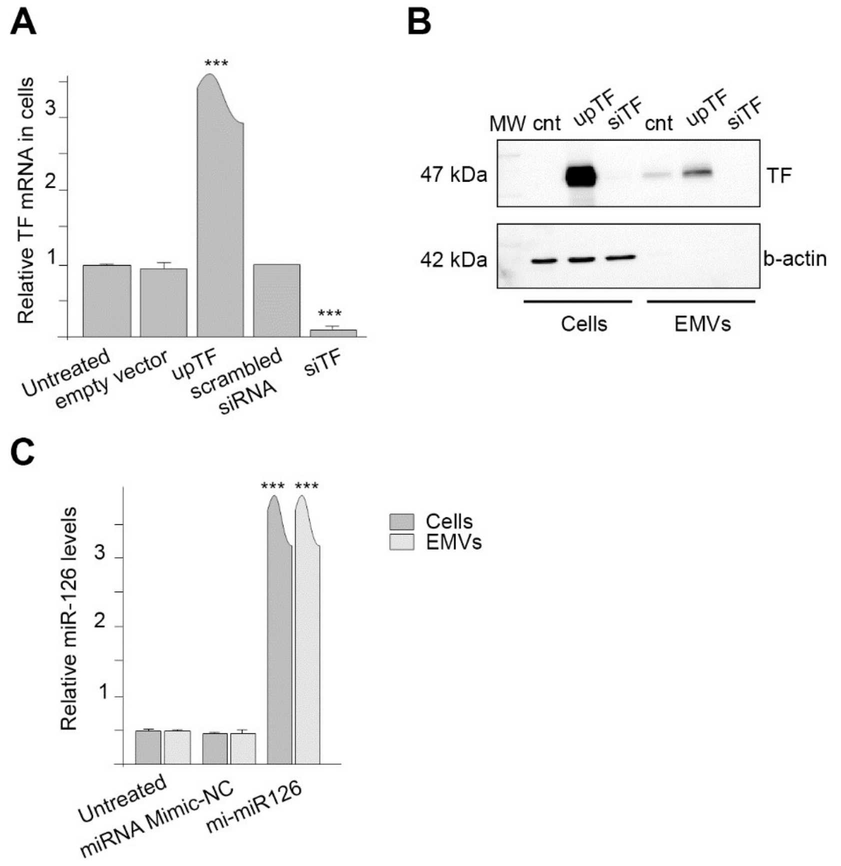

(A) TF mRNA levels in ECs transfected as indicated. Results are expressed as mean ± SEM, statistical analysis was performed by ANOVA followed by Tukey post hoc test. ***p<0.001 *versus* untreated (N=6). **(B)** TF protein expression in ECs and their MVs isolated from migrating endothelial cells transfected as indicated. Representative image from six independent experiments. **(C)** MiR-126 expression levels in ECs and their EMVs transfected as indicated. Results are expressed as mean ± SEM, statistical analysis was performed by ANOVA followed by Tukey post hoc test. ***p<0.001 *versus* untreated (N=6)

Supplementary Figure 2

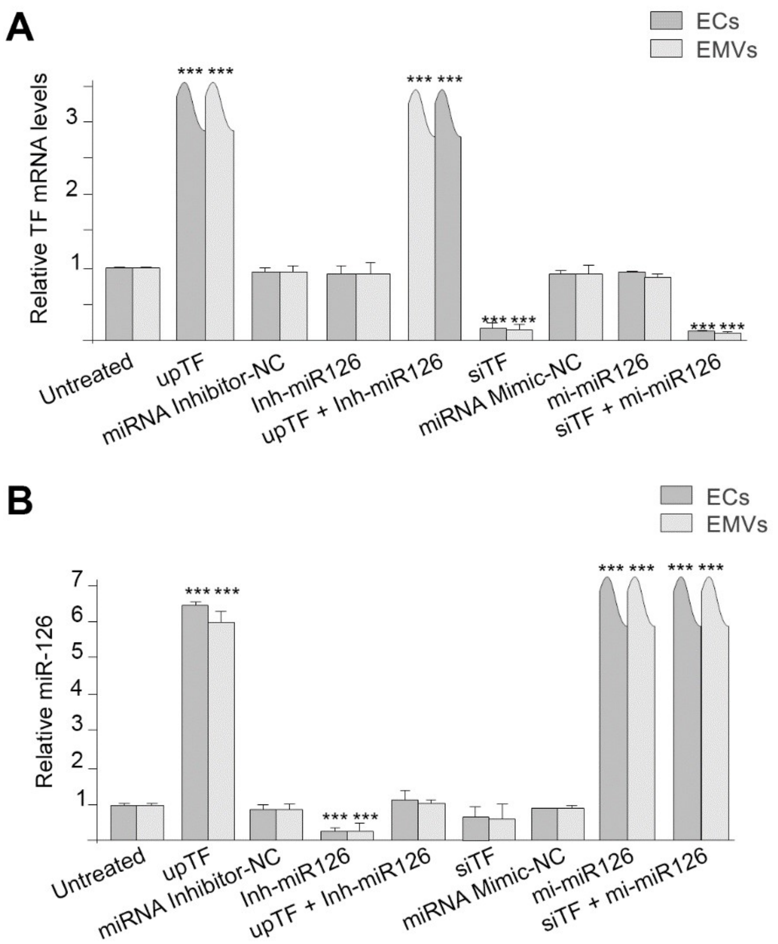

TF **(A)** and miR-126 **(B)** expression in ECs (dark grey) and their EMVs isolated (light grey) from migrating ECs transfected as indicated. Results are expressed as mean ± SEM, statistical analysis was performed by ANOVA followed by Tukey post hoc test. ***p<0.001 *versus* untreated (N=6)

Supplementary Figure 3

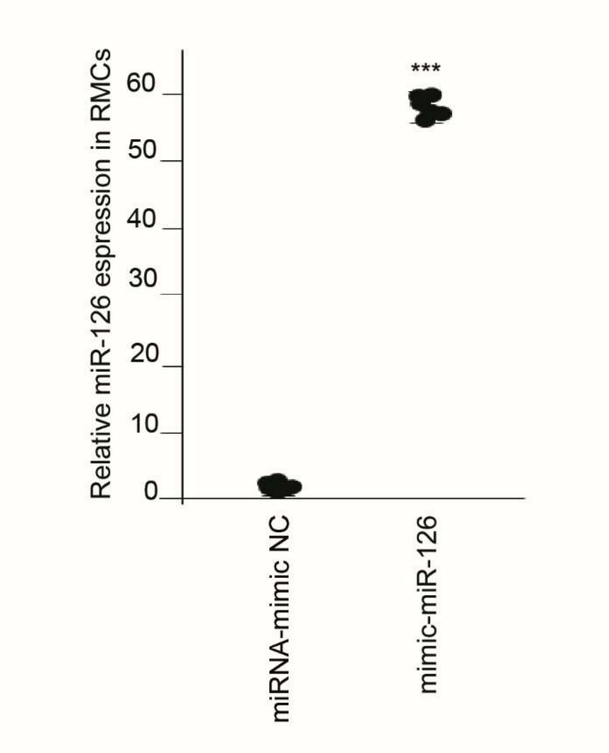

Expression of miR-126 in RMCs after transfection. Real time polymerase chain reaction analysis of miR-126 in RMCs transfected with miR mimic negative control (NC) or mimic –miR-126. Results are expressed as miR-126 relative levels ± SEM, statistical analysis was performed by ANOVA followed by Tukey post hoc test. ***$p<0.001$ (N=6).

Supplementary Figure 4

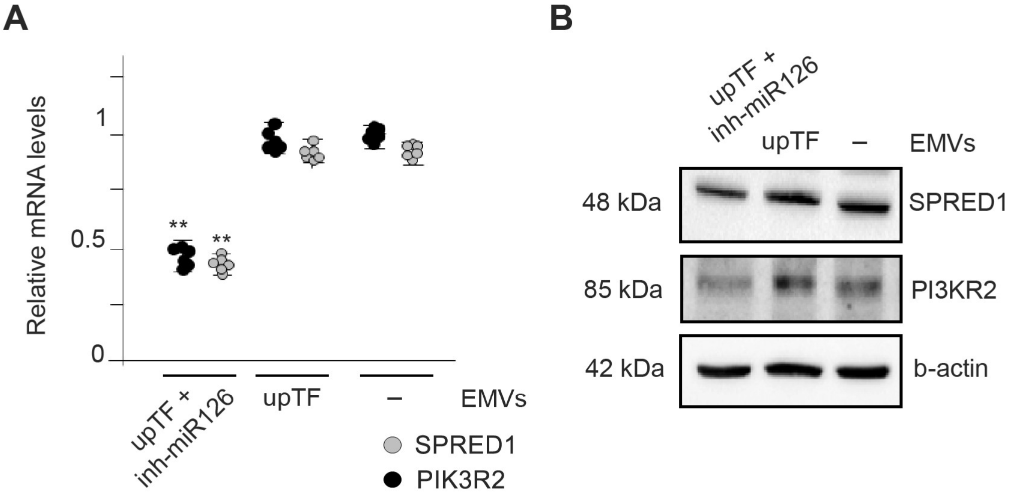

miR-126 and not TF regulates SPRED1 and PI3KR2 in RMCs. (A) Real time polymerase chain reaction analysis of SPRED1 and PI3KR2 in RMCs treated with EMVs obtained from ECs transfected with TF overexpression (upTF) and with or without (upTF + inh-miR126) expression of miR-126. Values are relative levels ± SEM, statistical analysis was performed by ANOVA followed by Tukey post hoc test. **p<0.01 *versus* RMCs treated with upTF-EMVs (N=6). **(B)** Western blot analysis of SPRED1 and PI3KR2 protein in RMCs treated with EMVs obtained from ECs transfected with TF overexpression (upTF) and with or without (upTF + inh-miR126) expression of miR-126. To test for equal loading westerns were re-probed by β-actin (N=6).
